# Impact of preoperative white blood cell count on outcomes in different stage colorectal cancer patients undergoing surgical resection: a single-institution retrospective cohort study

**DOI:** 10.1186/s12885-024-11983-7

**Published:** 2024-02-21

**Authors:** Bei Wang, Dandan Ling, Lihong Li, Jun Zhang, Jianghui Xu

**Affiliations:** 1 Department of Anesthesiology, Fudan University Shanghai Cancer Center, 200032 Shanghai, China; 2 Department of Oncology, Shanghai Medical College, Fudan University, 200032 Shanghai, China

**Keywords:** Colorectal cancer (CRC), Preoperative white blood cell (WBC) count, Overall survival (OS), Disease-free survival (DFS)

## Abstract

**Purpose:**

To explore the association between preoperative WBC count and the long-term survival outcomes and clinical outcomes in different stage patients who underwent surgical resection for colorectal cancer (CRC).

**Patients and methods:**

A cohort of 8121 Chinese patients who underwent surgical resection for CRC from January 1, 2008 to December 31, 2014 were enrolled as part of the retrospective cohort were retrospectively analyzed. Based on that the preoperative WBC optimal cut-off value was 7*10^9^/L (7,000/µL), the high preoperative WBC group and the low preoperative WBC group was defined. Inverse probability of treatment weighting (IPTW) using the propensity score was used to reduce confounding. The impact of preoperative WBC count on overall survival (OS) and disease-free survival (DFS) was investigated using the Kaplan-Meier method and Univariate Cox proportional hazards models in different stage subgroup respectively.

**Results:**

After IPTW, the clinical characters in the high preoperative WBC count group and the low preoperative WBC count group were balanced. Kaplan-Meier analysis showed that the 5-year OS rate were significantly lower in the high preoperative WBC count group overall, in stage II and IV. The 5-year DFS rate was significantly lower overall, in stage II and III in the high preoperative WBC count group. High preoperative WBC count was associated with poorer OS overall in stage II and stage IV.

**Conclusions:**

This study suggests that preoperative WBC count is an independent risk factor for survival in patients undergoing colorectal surgery and may need to consider the stage of cancer when applied to predict long-term adverse outcome prognosis.

## Introduction

Colorectal cancer (CRC) is the third most commonly diagnosed malignancy and the second leading cause of cancer death worldwide, with an emerging trend of the incidence of CRC at younger ages (before age 50 years) rising [1–3]. There were 376,300 cases and 191,000 deaths of CRC in China according to the statistics data in 2015 and there are estimated 592,232 cases and 309,114 deaths of CRC in China in 2022 [4, 5]. The global burden of colorectal cancer (CRC) is expected to increase by 60% to more than 2.2 million new cases and 1.1 million deaths by 2030 [6]. Reductions in colon and rectal cancer mortality rates are probably due to better accessibility to early detection services and improved specialized care [7]. Although diagnosis, treatment, radiotherapy, and chemotherapy have made great progress, surgery remains the primary treatment of choice. However, there are still many relapses and metastases occur [8]. We urgently require a diagnostic marker to predict the prognosis of CRC patients after surgery in different tumor-node-metastasis (TNM) stages.

Inflammation can be one of the underlying mechanisms linking lifestyle to fatigue in CRC and others cancer patients [9–12]. CRC is a tumor closely associated with inflammation, where heterogeneous immune cell infiltration and peripheral hematologic features disorders affect the complex microenvironment that allows tumor development [13, 14].

Preoperative hematological markers, as routine preoperative tests, help to suggest the prognosis of cancer patients [15–17]. The correlation between high WBC count and poor prognosis has been reported in, for example, oropharyngeal, cervical, esophageal anal cancers [18–21]. Recently few previous studies have suggested that preoperative leukocyte counts may predict the prognosis of patients undergoing CRC surgery [14, 22]. Patients at the same stage have different survival outcomes [23]. We noticed that there are significant differences in preoperative WBC count at different stages. However, previous retrospective analyses have been limited by small sample size, more confounding factors, short follow-up time, inability to cover patients at all stages after matching, and lack of stratified studies for different stages. In particular, whether the relationship between preoperative white blood cell count and prognosis may differ in the different stages of the patient has not been elucidated.

The purpose of this study was to further investigate the prognostic value of preoperative WBC count on overall survival and disease-free survival after surgery for colorectal cancer in a large retrospective cohort of Chinese patients, to retain more case data, and to perform an analysis of the relationship between the two at different tumor stages. We predicted that the relationship between preoperative WBC count and survival outcomes in CRC patients at different stage is different.

## Materials and methods

### Study design and study population

**Fig. 1:**
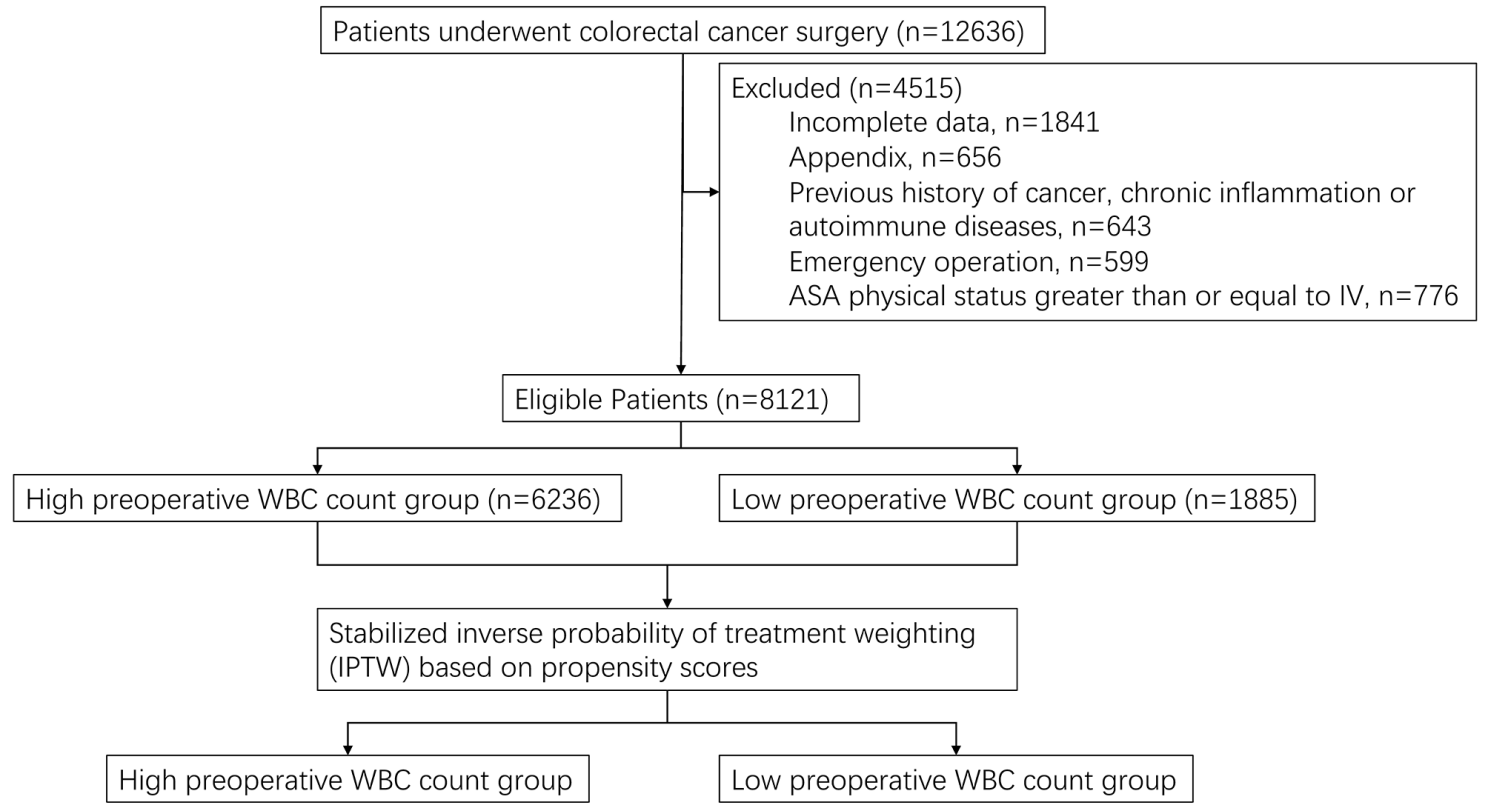
Flow chart of cohort study. WBC, white blood cell

This was a retrospective, observational study. 8121 Chinese patients in Fudan University Shanghai Cancer Center, Shanghai from January 1, 2008 to December 31, 2014 were enrolled as part of the retrospective cohort in (Fig. 1). The present study was approved by the Ethics Committee of Fudan University Shanghai Cancer (FUSCC) (No. IRB2105235-6), China. All methods in the study were carried out in accordance with relevant guidelines and regulations of the Declaration of Helsinki. The patients have signed informed consent in the study. Patients who were included underwent surgical resection for CRC with complete clinical history data, were followed up until death or December 31, 2019. The data were gathered from the database of the hospital clinical information system database. The medical information of each patient was recorded, including age and gender, medical history, pathological information, tumor pathological type, location, differentiation, stages I/II/III/IV (Tumors were staged according to the seventh version of the American Joint Committee on Cancer (AJCC) tumor–nodes–metastasis (TNM) classification), operative details, and postoperative outcomes. The eligibility criteria were as follows: complete medical history and follow-up data; elective surgery for CRC; no other synchronous malignancy. The exclusion criteria included incomplete data, previous history of cancer, chronic inflammation or autoimmune diseases, the surgery was emergency, and the American Society of Anesthesiologists (ASA) physical status greater than or equal to IV. Patients were followed up every 3 months for the first 2 years after surgery, every 6 months thereafter for 3 years and then every 1 year after 5 years. Abdominopelvic and chest computed tomography (CT) scans were performed every 6 months, while colonoscopy was performed annually.

### Exposure

The preoperative peripheral WBC count was collected from the blood routine examination tested by the instrument “The Sysmex XN-9000” within 5 days before surgery. According to the several previous studies, determination of the optimal cut-off value for preoperative WBC was performed by using X-tile 3.6.1 software (Yale University, New Haven, CT, USA) with the minimum *P*-values from log-rank×2 statistics for survival, which was 7*10^9^/L (7,000/µL) [14, 19, 22]. Based on this WBC cut-off value, the patients were divided into two groups, high preoperative WBC count group and low preoperative WBC count group.

### Outcomes and variables

The primary outcomes were overall survival (OS) and disease-free survival (DFS) after surgery in CRC patients at different stage. OS was defined as the interval between the date of diagnosis and the date of death from any cause, or the last follow-up date. DFS was defined as the interval between the date of diagnosis and the date of recurrence, metastasis, occurrence of a secondary primary tumor, death or the last follow-up date. The secondary outcomes were clinical parameters, including reoperation with in 30 days, the length of postoperative hospitalization, and intraoperative bleeding at different stage. We reviewed and recorded the following variables from the clinical information system of the hospital database: gender, age, preoperative adjuvant chemotherapy, surgical approach, tumor location, tumor pathological type, tumor differentiation, vascular tumor thrombus, perineural invasion, surgical margin positivity, surgical procedure, stages, number of cancer nodules ≥ 1. The work has been reported in line with the STROCSS criteria [24].

### Statistical analysis

The study was analyzed by SPSS software. All data were expressed as number (percentages) or the mean ± SD. The relationship between WBC count and the baseline parameters of clinical conditions was analyzed by chi-square tests.

In order to adjust for selection bias and potential confounding factors between patient groups in comparisons of outcomes, we performed stabilized inverse probability of treatment weighting (IPTW) based on propensity scores to control for differences in baseline characteristics between high preoperative WBC count and low preoperative WBC count patients. A logistic regression model was used to calculate propensity scores including the covariates: gender, treat year, age,stage, tumor location, tumor pathological type, tumor differentiation, vascular tumor thrombus, perineural invasion, incisal margin, surgical procedure, preoperative chemotherapy, cancer node. Standardized mean differences (SMD) were used to measure the balance of individual covariates before and after IPTW. Differences were considered statistically significant at SMD > 10%.

We use the Kaplan–Meier method to analyze survival rates. IPTW-adjusted Kaplan–Meier survival curves and the weighted log-rank test were generated by comparing the high preoperative WBC count groups. The effects of high preoperative WBC count and other potential prognostic factors presented as IPTW-adjusted hazard ratios (HRs) and 95% confidence intervals (CIs) were estimated using the weighted Cox proportional hazards model. If the Proportional Hazards Assumption does not hold during the analysis of the Cox Proportional Hazards Model, the stratified Cox model or the method of including time-dependent covariates for the analysis would be used. Hypothesis testing was performed at a two-sided 5% significance level. Statistical analyses were performed using R version 3.4.3 (R Foundation for Statistical Computing). We using the *t* test or χ2 test to compare the variables of clinical outcomes.

## Results

A total of 8121 Chinese patients with CRC undergoing elective surgical resection were enrolled in this study. All the patients were divided into two groups, the high preoperative WBC count group (WBC count ≥ 7,000/µL) and the low preoperative WBC count group (WBC count < 7,000/µL). The baseline characteristics are shown in Table 1. The incidence of high preoperative WBC count was 23.2% (1,885 out of 8,121 patients). Patients with high preoperative WBC count were more male (64.6% vs. 57.6%, *p* < 0.001), received less preoperative chemotherapy (5.7% vs. 9.1%, *p* < 0.001), more mucoid adenocarcinoma (14.3% vs. 11.1%, *p* < 0.001), signet cell cancer (1.9% vs. 1.4%, *p* < 0.001) and had less well differentiation tumors (1.5% vs. 2.3%, *p* < 0.001) than those with low preoperative WBC counts. The high preoperative WBC count group had more patients than the low preoperative WBC count group with poorer stage (IV 16.6% vs. 12.1%, *p* < 0.001), more patients with left colon (23.0% vs. 20.5%, *p* < 0.001), right colon (27.0% vs. 23.8%, *p* < 0.001) and transverse colon (1.6% vs. 1.2%, *p* < 0.001). Compared with low preoperative WBC count group, high preoperative WBC count group had less rectal location (48.2% vs. 54.2%, *p* < 0.001), surgical margin positivity (2.3% vs. 1.4%, *p* = 0.008). There was no significant difference in treat year (*p* = 0.916), age (*p* = 0.446), vascular tumor thrombus positivity (*p* = 0.111), perineural invasion positivity (*p* = 0.750), cancer node (n > = 1, *p* = 0.298) and open surgical procedure ( *p* = 0.298).

**Table 1:** Patients characteristics of the high preoperative WBC count group and the low preoperative WBC count group before and after IPTW. WBC, white blood cell; IPTW, inverse probability of treatment weighting

Variables	Entire study population				Weighted covariates			
	Low pre-WBC count *n* = 6236	High pre-WBC count *n* = 1885	*P* value	Standardized difference (%)	Low pre-WBC count *n* = 6235.3	High pre-WBC count *n* = 1889.2	*P* value	Standardized difference (%)
Gender			< 0.001	0.144			0.808	0.007
Female	2642[42.4]	667[35.4]			2541.4[40.8]	776.2[41.1]		
Male	3594[57.6]	1218[64.6]			3693.9[59.2]	1113.0[58.9]		
Treat year			0.916	0.003			0.721	0.010
2008–2012	3902[63]	1182[63]			3906.0[62.6]	1192.3[63.1]		
2013–2014	2334 [37]	703 [37]			2329.3[37.4]	696.9[36.9]		
Age[years]			0.446	0.050			0.996	0.012
=<44	799[12.8]	269[14.3]			822.1[13.2]	252.2[13.3]		
45–54	1247[20.0]	386[20.5]			1253.2[20.1]	379.7[20.1]		
55–64	2263[36.3]	652[34.6]			2235.4[35.8]	670.4[35.5]		
65–74	1342[21.5]	404[21.4]			1340.6[21.5]	404.9[21.4]		
>=75	585[9.4]	174[9.2]			584.1[9.4]	182.0[9.6]		
Stage			< 0.001	0.218			0.992	0.014
I	1185[19.0]	259[13.7]			1109.6[17.8]	339.6[18.0]		
II	1665[26.7]	578[30.7]			1722.1[27.6]	519.7[27.5]		
III	2445[39.2]	707[37.5]			2419.4[38.8]	729.7[38.6]		
IV	756[12.1]	313[16.6]			820.4[13.2]	246.6[13.1]		
Unknown	185[3.0]	28[1.5]			163.9[2.6]	53.5[2.8]		
Tumor location			< 0.001	0.120			0.978	0.012
Rectal	3381[54.2]	909[48.2]			3295.9[52.9]	1008.0[53.4]		
Left colon	1283[20.6]	433[23.0]			1316.1[21.1]	392.5[20.8]		
Right colon	1560[25.0]	539[28.6]			1611.0[25.8]	484.5[25.6]		
Total colon	12[0.2]	4[0.2]			12.4[0.2]	4.2[0.2]		
Tumor pathological type			< 0.001	0.109			0.987	0.004
Adenocarcinoma	5460[87.6]	1579[83.8]			5403.7[86.7]	1635.3[86.6]		
Mucoid adenocarcinoma	690[11.1]	270[14.3]			737.6[11.8]	224.6[11.9]		
Signet cell cancer	86[1.4]	36[1.9]			94[1.5]	29.3[1.6]		
Tumor differentiation			< 0.001	0.130			0.959	0.016
Poor	1229[19.7]	452[24.0]			1291.2[20.7]	392.1[20.8]		
Moderate	4233[67.9]	1254[66.5]			4211.5[67.5]	1267.7[67.1]		
Well	143[2.3]	29[1.5]			132.5[2.1]	43.8[2.3]		
Unknown	631[10.1]	150[8.0]			600.1[9.6]	185.6[9.8]		
Vascular tumor thrombus			0.118	0.042			0.872	0.004
Yes	1402[22.5]	457[24.2]			1426.1[22.9]	428.7[22.7]		
No	4834[77.5]	1428[75.8]			4809.2[77.1]	1460.5[77.3]		
Perineural invasion			0.776	0.008			0.898	0.003
Yes	1187[19.0]	365[19.4]			1192.1[19.1]	358.7[19.0]		
No	5049[81.0]	1520[80.6]			5043.2[80.9]	1530.5[81.0]		
Surgical margin positivity			0.011	0.065				
Yes	90[1.4]	44[2.3]			102.9[1.7]	30.9[1.6]	0.964	0.001
No	6146[98.6]	1841[97.7]			6132.4[98.3]	1858.3[98.4]		
Surgical procedure			0.794	0.008			0.882	0.004
Open	5737[92.0]	1730[91.8]			5733.1[91.9]	1735[91.8]		
Video-assisted	499[8.0]	155[8.2]			502.2[8.1]	154.2[8.2]		
Preoperative chemotherapy			< 0.001	0.128			0.700	0.012
Yes	566[9.1]	108[5.7]			518.5[8.3]	163.4[8.6]		
No	5670[90.9]	1777[94.3]			5716.8[91.7]	1725.8[91.4]		
Cancer node			0.331	0.026			0.977	0.001
>=1	946[15.2]	304[16.1]			959[15.4]	291.4[15.4]		
0	5290[84.8]	1579[83.9]			5276.3[84.6]	1597.8[84.6]		

IPTW was used to reduce the imbalance in baseline characteristics between the high preoperative WBC count group and the low preoperative WBC count group. After IPTW, the clinical characters in two groups were all balanced (Table 1).

**Fig. 2:**
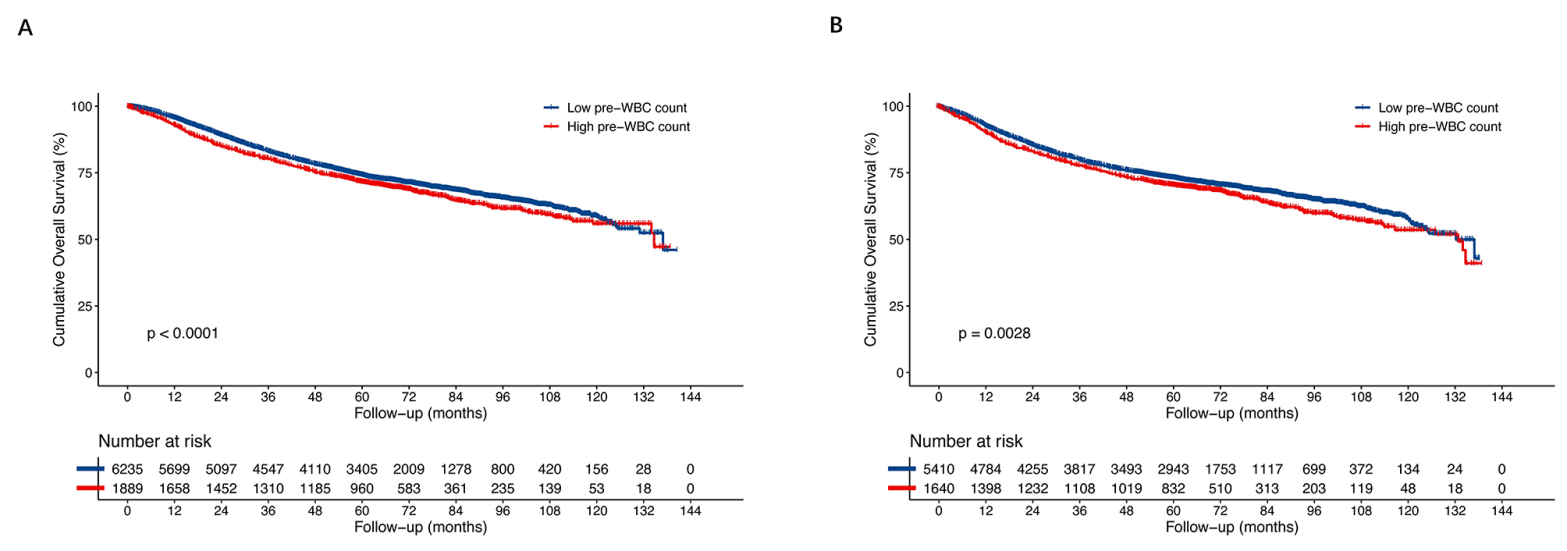
Overall survival (OS) and disease-free survival (DFS) after weighting by the Kaplan–Meier method and the weighted Cox proportional hazards model after IPTW. **A** The 5-year OS rate was significantly worse in the high preoperative WBC group than in the low preoperative WBC group (71.6% vs. 74.3%, *p* < 0.0001); **B** The 5-year DFS rate was significantly worse in the high preoperative WBC group than in the low preoperative WBC group (70.5% vs. 73.2%, *p* = 0.0028). OS, overall survival; DFS, disease-free survival; IPTW, inverse probability of treatment weighting; WBC, white blood cell

After IPTW, the Kaplan–Meier analysis showed that the 5-year OS rate (71.6% vs. 74.3%, *p* < 0.0001) was significantly lower in the high preoperative WBC counts group than in the low preoperative WBC counts group (Fig. 2). Overall, the 5-year DFS rate (70.5% vs. 73.2%, *p* = 0.0028) was lower in the high preoperative WBC counts group than in the low preoperative WBC counts group (Fig. 2).

**Table 2:** Clinical outcomes of the high preoperative WBC count group and the low preoperative WBC count group before and after IPTW. WBC, white blood cell; IPTW, inverse probability of treatment weighting

	Before IPTW			After IPTW		
	Low pre-WBC count *n* = 6236	High pre-WBC count *n* = 1885	*P* value	Low pre-WBC count *n* = 6235.3	High pre-WBC count *n* = 1889.2	*P* value
Re-operation with in 30 days			0.860			0.829
Yes	113[1.8]	33[1.8]		113.493[1.8]	32.959[1.7]	
No	6123[98.2]	1852[98.2]		6121.825[98.2]	1856.220[98.3]	
Number of days in hospital(days)	18.25 ± 0.29	18.10 ± 0.17		18.27 ± 0.30	18.17 ± 0.16	0.86
Intraoperative bleeding(mL)	74.81 ± 1.09	79.05 ± 1.65		75.03 ± 1.11	78.02 ± 1.61	0.17

In the weighted Cox proportional hazards model, on univariate analysis of survival, high preoperative WBC count was associated with a strong trend toward worse OS (HR 1.14; 95% CI 1.034–1.256; *p* = 0.008). As for DFS, the presence of high preoperative WBC count was a significant factor in the univariate analysis (HR1.143; 95% CI 1.031–1.267; *p* = 0.011) (Table 3).

**Table 3:** Univariate analysis for overall survival and disease-free survival after IPTW. WBC, white blood cell; IPTW, inverse probability of treatment weighting

		Univariate analysis	
		HR (95% CI)	*p* value
Overall survival			
ALL	Low pre-WBC count	1(reference)	0.008
	High pre-WBC count	1.335(1.205, 1.480)	
TNM I	Low pre-WBC count	1(reference)	0.981
	High pre-WBC count	1.005(0.696, 1.452)	
TNM II	Low pre-WBC count	1(reference)	0.023
	High pre-WBC count	1.309(1.036, 1.653)	
TNM III	Low pre-WBC count	1(reference)	0.069
	High pre-WBC count	1.147(0.994, 1.324)	
TNM IV	Low pre-WBC count	1(reference)	0.002
	High pre-WBC count	1.302(1.100, 1.540)	
Disease-free survival			
ALL	Low pre-WBC count	1(reference)	0.011
	High pre-WBC count	1.143(1.035, 1.263)	
TNM I	Low pre-WBC count	1(reference)	0.956
	High pre-WBC count	1.009(0.749, 1.360)	
TNM II	Low pre-WBC count	1(reference)	0.018
	High pre-WBC count	1.259(1.04, 1.525)	
TNM III	Low pre-WBC count	1(reference)	0.038
	High pre-WBC count	1.15(1.008, 1.312)	

**Fig. 3:**
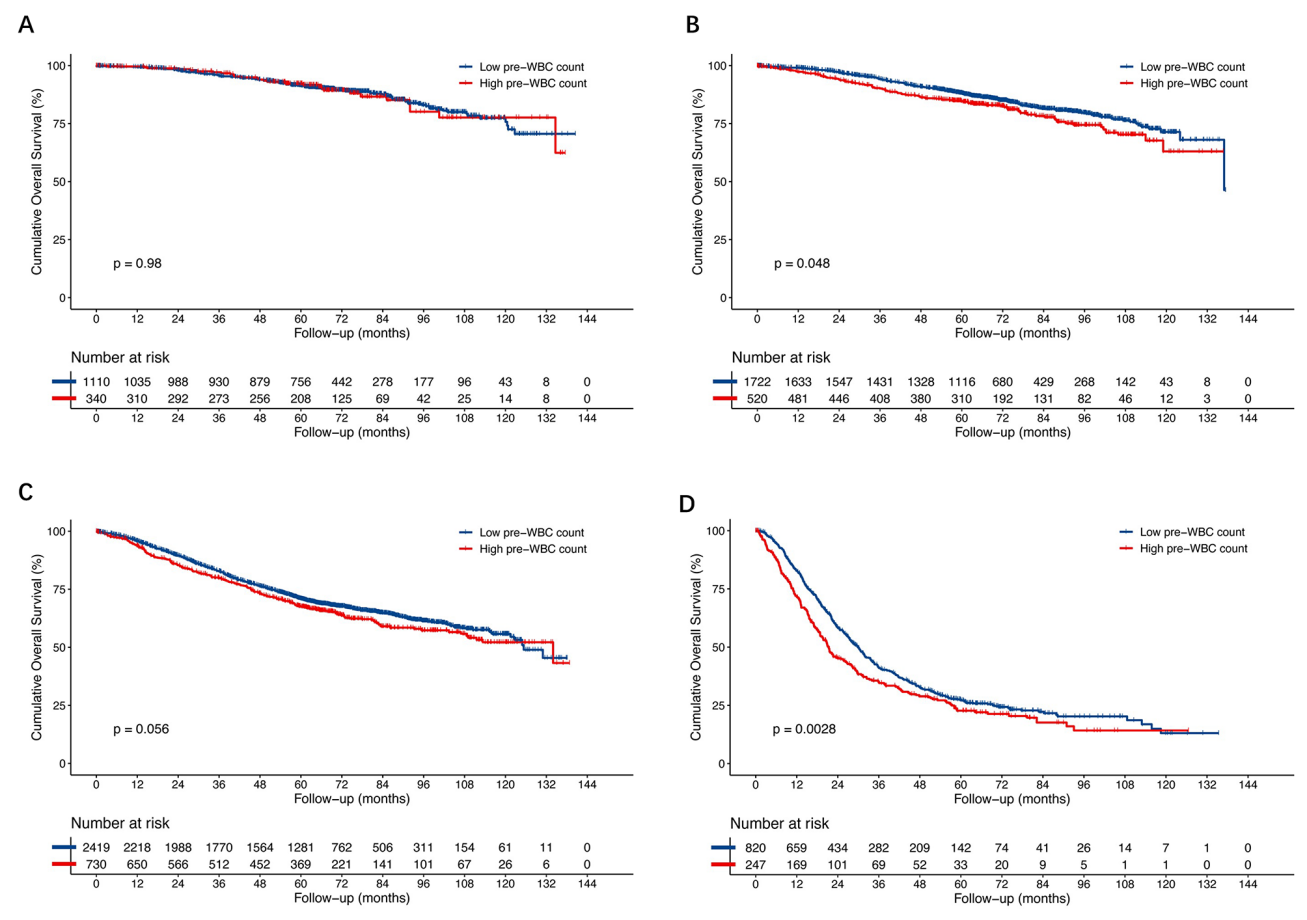
Overall survival (OS) after weighting by the Kaplan–Meier method stratified by stage after IPTW. **A** stage I (5-year OS rate 91.5% vs. 92.3%, *p* = 0.098). **B** stage II (5-year OS rate 84.7% vs. 88.2%, *p* = 0.048). **C** stage III (5-year OS rate 71.0% vs. 67.6%, *p* = 0.056). **D** stage IV (5-year OS rate 22.7% vs. 27.4%, *p* = 0.028) (Fig. 3). OS, overall survival; IPTW, inverse probability of treatment weighting; WBC, white blood cell

To investigate the impact of preoperative WBC count on survival in patients of each stage, we used a stratified analysis. After IPTW, the Kaplan–Meier analysis showed that the 5-year OS rate were significantly lower in the high preoperative WBC count group in stage II (84.7% vs. 88.2%, *p* = 0.048) and stage IV (22.7% vs. 27.4%, *p* = 0.028), but not in stage I (5-year OS rate 91.5% vs. 92.3%, *p* = 0.098) or stage III (5-year OS rate 71.0% vs. 67.6%, *p* = 0.056) (Fig. 3).

**Fig. 4:**
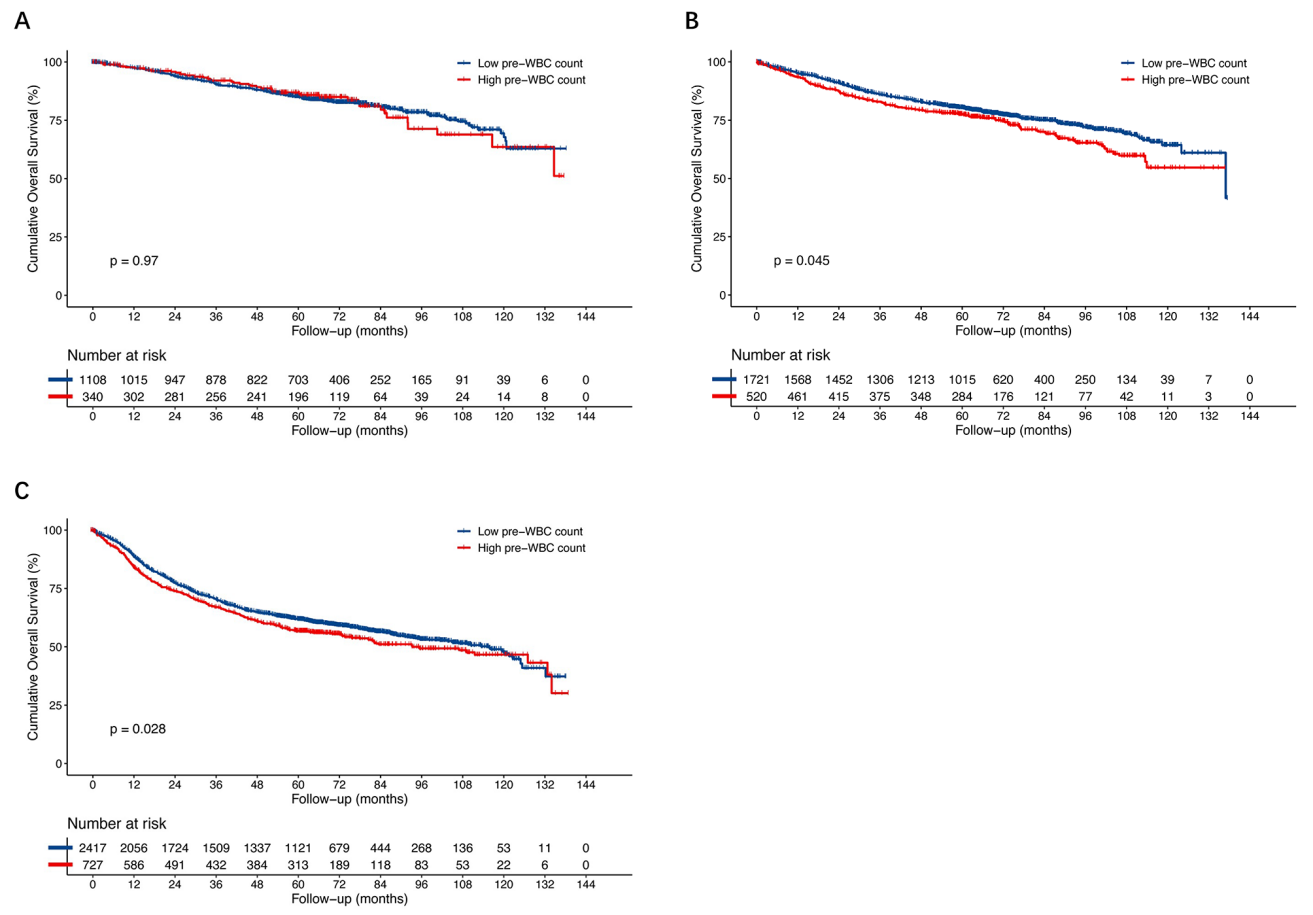
Disease-free survival (DFS) after weighting by the Kaplan–Meier method stratified by TNM stage after IPTW. **A** stage I (5-year DFS rate 85.2% vs. 86.9%, *p* = 0.97). **B** stage II (5-year DFS rate 77.6% vs. 80.4%, *p* = 0.045). **C** stage III (5-year DFS rate 56.8% vs. 61.9%, *p* = 0.028) (Fig. 4). DFS, disease-free survival; IPTW, inverse probability of treatment weighting; WBC, white blood cell

IPTW analysis showed that the 5-year DFS rate were significantly lower in the high preoperative WBC count group in stage II (77.6% vs. 80.4%, *p* = 0.045) and stage III (56.8% vs. 61.9%, *p* = 0.028), but not in stage I (5-year DFS rate 85.2% vs. 86.9%, *p* = 0.97) (Fig. 4).

In the weighted Cox proportional hazards model in different stage subgroup, the association between preoperative WBC count and survival showed different. In univariate analysis of survival, high preoperative WBC count was associated with a strong trend toward worse OS (HR 1.14; 95% CI 1.034–1.256; *p* = 0.008) and DFS (HR1.143; 95% CI 1.031–1.267; *p* = 0.011) (Table 3). In the analysis of the overall survival in the propensity after IPTW, the association between preoperative WBC count and overall survival is significantly different in stage II (HR 1.309; 95%CI 1.036–1.653; *p* = 0.023) and stage IV (HR 1.302; 95%CI 1.100–1.540; *p* = 0.002), but not in stage I (HR 1.005; 95%CI 0.696–1.452; *p* = 0.981) and stage III (HR 1.147; 95%CI 0.994–1.324; *p* = 0.069). In the analysis of the DFS in the propensity after IPTW, the association between preoperative WBC count and overall survival is significantly different in stage II (HR 1.259; 95%CI 1.04–1.525; *p* = 0.018) and stage III (HR 1.148; 95%CI 1.008–1.312; *p* = 0.038), but not in stage I (HR 1.009; 95%CI 0.749–1.360; *p* = 0.956) (Table 3).

In the analysis of postoperative recovery outcomes after IPTW, the 30-day re-operation rate, number of days in hospital and volume of intraoperative bleeding were not significantly different between two groups (Table 2).

## Discussion

It is a retrospective, real-world, follow-up cohort-base study. As the anesthesiologists in China, we care for the patients not only shot-term outcome, but also long-term outcome. We hope that the cancer patients have a comfortable experience perioperatively and better outcome as well. Therefore, we focused on 8121 Chinese CRC patients during a six-year time period. In the present study, the impact of preoperative WBC count as a prognostic factor was investigated to predicting survival in patients with CRC. We confirmed that high preoperative WBC count had a poorer OS and DFS and explored the relationship between preoperative WBC count and prognosis in different stages in this study.

Some studies have demonstrated that peripheral blood leukocytosis and neutrophilia reflected cancer-related inflammation and has been proposed as prognostic immunological biomarkers for various malignancies [11, 25]. A study validated leukocytosis as an independent prognostic factor in CRC, which provided for the first-time vital insight on the correlation of peripheral pretreatment leukocytosis with the tumor-infiltrating cells contexture and might be relevant for future risk stratification [14]. A meta-analysis showed that preoperative leukocytosis was common and correlates with poor pathological and survival outcomes in endometrial carcinoma patients [26]. A study showed that preoperative leukocytosis and the resection severity index were independent risk factors for survival in patients with intrahepatic cholangiocarcinoma [27]. Cancer patients with acute venous thromboembolism and elevated WBC count had an increased incidence of VTE recurrences, major bleeding, or death [28]. In a retrospective analysis concerning cervical cancer, patients with leukocytosis (WBC ≥ 10,000/µL) showed significantly higher treatment failure rate (*P* < 0.001) and shorter OS (*P* < 0.001) than the patients without leukocytosis. In a prospective investigation, patients with leukocytosis exhibited a significantly higher treatment failure rate (*P* < 0.001), shorter PFS (*P* < 0.001) than did the patients without leukocytosis [18]. A study showed that preoperative asymptomatic leukocytosis had a prevalence of 5.6% in CRC resections and carried a significant increased risk of mortality and morbidity [29].An increasing body of evidence supports that visibility of CRC to immune attack is substantial and that it limits disease progression. Analysis of the adaptive immune infiltrate in resected CRC specimens offers prognostic information which is independent of conventionally measured parameters and potentially superior in predictive value [30]. A high WBC and lymphocyte count combined with normal testosterone levels increases the overall mortality of patients treated with radiotherapy for localized prostate cancer within the first 6–7 years post-treatment [31]. Leukocyte and neutrophil count parameters might be clinically relevant biomarkers to be considered for further clinical investigations [32]. Gaining a better mechanistic understanding of the mode of action of anti-inflammatory agents and designing more effective treatment combinations would advance the clinical application of this therapeutic approach [33]. A retrospective cohort study showed that treatment-related leukopenia in anal cancer patients was associated with worse outcome [34]. Further subgroup analysis indicated that preoperative moderate leukocytosis was significantly associated with poorer OS and DFS in patients with no preoperative chemotherapy. Despite numerous reports detailing the interplays between cancer and its microenvironment via the inflammatory network, the status of cancer-associated inflammation remains difficult to identify in clinical settings [35]. Systemic inflammation is preoperatively a marker indicating poor prognosis, which is present in approximately 20-40% of CRC patients [36]. A study suggested that the leukocyte and neutrophil count parameters may be clinically relevant biomarkers; therefore, further clinical investigations are required [32]. It was found that tumor-infiltrating lymphocyte counts are associated with peculiar gene expression patterns and bear prognostic information in ovarian cancer [37]. The meta-analysis showed that preoperative neutrophil-to-lymphocyte ratio is an independent risk factor for poor prognosis in patients with colorectal liver metastasis [38].

Nevertheless, patients may have different long-term outcomes even at the same stage [23]. In terms of value for clinical application, the value of stage II may be more significant than that of stage I. We suggest that the application of preoperative WBC count as a clinical indicator for prediction may require reference to the stage. So, it is possible that the immune status varies from different stage and thus has an impact on prognosis, which of course requires further prospective clinical trials and mechanistic studies [39]. It has been shown that using the immunoscore for cancer can be used as a reference for the prognosis of tumor patients [40–43]. However, immunoscore is often performed postoperatively, and it is difficult to predict survival preoperatively. As part of the manifestation representing the immune status of the tumor, preoperative WBC count have a predictive role and can predict more easily and quickly. Preoperative leukocytes are more predictive in stage II patients and may be related to the immune status of stage II patients, and more further studies are needed, while preoperatively prompting anesthesiologists and surgeons to pay more attention to patients with a higher inflammatory status in stage II and to improve the prognosis by taking measures to suppress the preoperative inflammatory status for the benefit of the patient.

To our knowledge, this study is the first to explore the relationship between WBC count and outcome for different stages. We found a nonsignificant association between high preoperative WBC and prognosis in stage I and III patients. We not only balanced the confounding factors by IPTW to ensure maximum statistical efficacy, but also stratified patients for personalized treatment. The present results provide evidence and a better understanding of relationship between different preoperative WBC count and prognosis of patients at different stages. However, our study still has a few limitations. It was retrospective and was not prospective or randomized, postoperative treatment heterogeneity was inevitable due to the retrospective design, which might affect our results. Though OS is considered the gold standard end-point in the aspect of cancer prognosis study, we lacked with relapse-free survival data and disease-specific survival data. Even if possible cofounding factors are eliminated, there are various factors affecting the preoperative white blood cell count, and there are some cofounding factors that are not measured and eliminated. Further clinical prospective studies and laboratory researches are needed to determine the mechanism of relationship between white blood cell count and outcome in patients undergoing surgery for CRC.

In conclusion, the high preoperative WBC count was significantly associated with survival in CRC negatively. High preoperative WBC count is associated with an increased risk of long-term adverse outcomes in CRC prognosis, with different strength of association for different stage. This study suggests that preoperative WBC count may need to take into account the stage of the cancer when applied to predict long-term adverse outcome.

## Data Availability

The datasets used and analysed during the current study available from the corresponding author on reasonable request.
